# Valedictory Address, Tenth Annual Commencement (Dental Dept.) State University of Iowa

**Published:** 1892-06

**Authors:** J. J. R. Patrick


### VALEDICTORY ADDRESS.

DELIVERED BY

J. J. R. PATRICK, D.D.S., AT THE

### Tenth Annual Commencement of the Dental Department, State University of Iowa, Thursday Evening, March 10th, 1892.

Impressed by the splendor which surrounds me, and by the sight of this large audience, I feel impelled to render homage to the beauty and intelligence which have prepared so brilliant a reception for the graduating class of the Dental Department of Iowa's University. If all the other branches of learning in the University have been as well cared for as the Dental Department, the Board of Regents, the Presiding Officer, and the Professors have reason to congratulate themselves.

The diffusion of knowledge has ever been considered one of the most important elements of the greatness of nations; all educational institutions are conducted in the interest and for the ultimate benefit of the public; but a State University is one that appeals more directly to the general interest, inasmuch as the influence of a University reaches far beyond the boundaries of State lines, and every graduate who goes out into the world is a qualified instructor in his every-day intercourse with his fellow-man, in some department of useful knowledge. It is not more than fifty years ago that this beautiful and fertile State of Iowa was the abode of savages and wild animals; large herds of buffalo, elk and deer roamed at will over the vast prairies, while more savage animals lurked in the boundless forests. About this time a stream of civilization commenced to flow from the older States which rolled down from the summits of the Alleghany Mountains and overspread the banks of the Ohio, the Mississippi, the Arkansas, and their tributary streams. This ceaseless flood of human beings continued to flow from the abodes of civilization, spreading out over an extent of nearly 1,200 miles in length and at an average advance of settlement and cultivation of seventeen miles a year. They did not come as the Tartar hordes, like a desolating fire or a raging torrent to oppress and overwhelm the opulent regions of the earth, but to assert their destined superiority over those of nature, to "replenish the earth and to subdue it." These early pioneers brought with them their own domestic animals, their agricultural and other implements, their seed grain, their powder and their rifles. They settled where they took up their abode, never to return. This tide of American population, streaming westward, was the wonder of the world, overspreading the wide fallow of the prairie till mile by mile the shore of the great ocean of human industry was lifted nearer and nearer to the distant sunset. But a time came at last when the wonder ceased. A fierce storm fell upon the central waters, the wave at the western margin no longer rippled further up the beach, but was sucked back, as it were, in sympathy with the distant tumult. The tide had turned and the current poured in dark and unwonted channels, a new emigration commenced—another goal was opened for the country's surplus life. The wagon, whose white canvass glittered like a sail, on many an occidental highway, was changed to an ambulance, or was laden with the stores of a quarter-master, the beast of burden labored as before, the farmer and his boy were still upon the march but it was the gun and the bayonet, and not the axe, that glittered above their shoulders and they rushed to fields where the harvest was red and not golden, where the reaper was death, and in the furrows of the field they won, many of those militant husbandmen found their last rest. The graves in the vast cemeteries of the nation are not altogether ghastly; a sacred pride goes hand in hand with grief to visit them, and glory and sorrow will ever sit at the tomb of those who prove that it is sweet to die for one's country. At the close of the war nearly 2,000,000 disciplined men returned to their homes and former avocations, or sought new ones. New cities came into existence and old ones increased in population. Locomotion, illumination, and the transmission of intelligence; in fact, every industry or occupation in life sprang forward and moved with accelerated speed. The discipline necessary to hold together such large bodies of men during the rebellion proved to be one of the principal factor's in the country's material progress. It was a school of instruction on a stupendous scale, the influence of which will reach to future generations. Verily "there's a divinity that shapes our ends, rough-hew them how we will." A further explanation of so rapid a progress is to be found in the constantly increasing division and subdivision of labor, building up the social fabric in the same manner that special cells compose and build up each part of a living organism; and it is a wise provision in nature that each division and subdivision of labor, each segmentation in the cellular formation of living organisms, is dependent on the others and is the outgrowth of natural laws. If we review the history of the sciences, we will find that marked advance has been made in a given science only when it has been connected with the progress of some collateral science or art. Thus the astronomer when connected with the optical instrument maker, became capable of rapid progress. The triumphs of magneticism and electricity, when the machinist and theorist were brought together, were rendered possible. To show how much we are indebted to the refinements of chemical analysis for our knowledge of the elementary constitution of bodies, and the great power it has given to man in his struggle towards civilization, would take volumes to recount. Two instances will suffice: The chemical combination of nitre, sulphur, and charcoal, known as gunpowder, and the reduction of iron from its ores. What changes they have wrought in the destiny of man! The bow and arrow would never have been effective in driving the wild animals from the prairies and out of the forests. The Saxon and his rifle made it possible for men to become skillful agriculturalists on a larger scale than was ever known before. No savage lives in harmony with the laws of nature. On the contrary, it is his ignorance of these laws that makes him a savage. And it is only in proportion as a man understands his surroundings--understands the laws that govern his existence and uses them to his advantage, and in that proportion only does he become civilized. The superiority of the civilized over the savage is in proportion to the extent to which his thought can grasp the past and the future. By his knowledge of letters he is enabled to record events which otherwise would escape his memory, and thus he augments his experience and frees himself from the inaccuracies of tradition. He realizes that it is not altogether what he sees, but how he sees. The senses, he has learned, are merely the substructure for the superstructure of thought. In uncultivated minds; the senses are the leaders. In cultivated minds, they are led by the understanding. A weak but disciplined eye can see more accurately than a strong, but undisciplined one. It discovers truth from many sides—is never thrown out of focus by chimeras, but receives the impress of objects truthfully; or, if the eye is defective by nature or enfeebled by age, the understanding corrects the error and restores the enfeebled eye to usefulness. When error is known truth is discovered. Follow your senses and they will lead you into a ditch; understand and cultivate them, and they will guide you in the paths of truth. I have been led to these reflections from having been a resident of the Territory of Iowa before it became a State, when the channels of industry were few and when a surplus of production was without a market. Journeys to any considerable distance could only be accomplished by the water highways. To leave Keokuk by boat, descend the Mississippi to the mouth of the Ohio, ascend the Ohio to Pittsburg, then by the old stage to Philadelphia over the mountain roads were an affair of such moment as seldom to be undertaken twice. Months scarcely sufficed to perform the journey; it was like a pilgrimage to Palestine in the Middle Ages. Now the distance is traversed in thirty hours. Among the great luxuries of the present age, the facilities for traveling are perhaps the most important, for they not only bring remote regions of the earth into proximity, but actually in a sense, bridge over the great gulf of time.

Ladies and Gentlemen of the Graduating Class: "The Dental Faculty of the University of the State of Iowa has assigned to me the duty to bid you on their behalf, an affectionate farewell.

The relation of pupil and teacher must now cease. The ties which have been formed amid the labors of the lecture room and made dear by years of pleasing associations, must now be broken. You have received, as evidence to the world, of your competency to practice your chosen profession, and as the reward of your industry, the degree of Doctor of Dental Surgery, and all the privileges pertaining to that degree. And you are now about to leave the walls of your *Alma Mater* to go out into the great world and engage in the practical duties of your profession. The occasion demands that I should present a few reflections for your consideration.

The honor conferred upon you this evening must receive additional value in your estimation, when you reflect that it has not been conferred upon you by an institute owned and conducted by a Joint Stock Company. On the contrary, it is your privilege, to have become the Alumni of the Dental Department of a great State University, formed by legislative enactment to carry out all the requirements relating to education; endowed by the State and General Government with vested rights, that it may endure forever.

You have now arrived at that period of your life when collegiate discipline has ceased to control your actions. Your researches in professional knowledge will no longer be subject to the direction of preceptors. You will go out into the world to mingle in the great contests of society, in honorable competition. Each will play his part and be the architect of his own fortune.

It is often the case, unfortunately, when students are released from the restrictions of college rules and have closed the period of their University exercises and taken their stations in life, that they relax their energies and become indifferent to scientific pursuits. Nothing can be more injurious to the best interests of society, or to your own spiritual health; nothing could be more disrespectful to your instructors and the good name of the institution that has vouched for your proficiency. In the bosom of every truly professional lady and gentleman there is an intense yearning for the acquisition of scientific knowledge, and if he or she has not this love abiding in the heart, they owe it to themselves, to the profession and to society, to abandon a profession they so unworthily esteem. The profession you have embraced and the honors you have earned and that have been conferred on you, this evening, have been conferred with the expectation that you will, from this time on, until life shall close, or until you shall relinquish it for some other vocation, devote your whole time and energy to the alleviation of human suffering, and thus, by the "relief of man's estate" earn for yourselves the title of benefactors of mankind. In order that you may become accomplished in your profession it will be necessary for you to devote your whole time and energy to it, irrespective of every disturbing temptation, for you should understand that your attendance on the prescribed course of lectures together with the ordinary pupilage in the laboratory, dissecting and clinic rooms, are not sufficient to make you accomplished dentists—all that can be taught or expected from a medical or dental school consists in a knowledge of those great general principles which are contained in the different departments of medical and dental science, and which are to be investigated and elaborated by yourself. What you have learned is all that can be taught—the foundation of your professional knowledge, upon which you are to rear the superstructure by constant study and clinical observation. A knowledge of your profession is of the first importance. But it is not all. You must practice what you know and practice in such a manner that it will be a blessing to the community in which you reside, while a source of emolument to yourself. A practice, however, must first be obtained, and the only way to obtain the patronage of a community is to merit it. It is unnessary for me to give in detail, the means by which you might secure a lucrative practice, by accommodating yourselves to the humors and idiosyncracies of a community in which you may cast your lot. Be careful, however, how you "stoop to conquer." It is a dangerous experiment at best, for what one individual would esteem politeness, another would denounce as obsequiousness. Your professional popularity will depend upon your personal popularity as well as upon your abilities; and your firmness of purpose and kindly feeling under the most trying circumstances in the discharge of your professional duties will secure both. As the smallest number of mankind are rich, and the largest may be divided into those in moderate circumstances, and those who are poor, and as all are subject to disease or accident, it follows that a doctor's fees are governed by these conditions. Discrimination in fees is therefore constant and necessary, whereas discrimination in merchandising, if indulged in to any extent, would be ruinous. A doctor's first care is the good of his patients, regardless of fees. A merchant's first care is his cash book. A doctor is no more legally bound to give his services for nothing than a merchant is to give his merchandise, but public opinion—stronger than statute law—says a doctor who can help a poor man and will not, without a fee, has less humanity than a poor ruffian who robs and maims a rich man to supply necessities. Public opinion says with truth it is something monstrous to contemplate a man of liberal education tearing out the bowels of a poor family, by taking for the services of one hour what would keep them in food for one week. This is why public opinion calls medicine, with all its specialties, a liberal profession. As you wish the respect of mankind, I charge you not to make a trade of your profession.

I can do no better than to commend to you the advice of Polonius to his son, Laertes, at parting:

> * * * * * * Give thy thoughts no tongue
> Nor any unproportioned thought his act.
> Be thou familiar, but by no means vulgar.
> The friends thou hast and their adoption tried
> Grapple them to thy soul with hoops of steel;
> But do not dull thy palm with entertainment
> Of each new hatched, unfledged comrade. Beware
> Of entrance to a quarrel, but being in,
> Bear it that the opposer may beware of thee.
> Give every man thine ear, but few thy voice;
> Take each man's censure, but reserve thy judgment.
> Costly thy habit as thy purse can buy.
> But not expressed in fancy; rich, not gaudy;
> For the apparel oft proclaims the man;
> And they in France of the best rank and station
> Are of a most select and generous chief in that.
> Neither a borrower nor a lender be;
> For loan oft loses both itself and friend;
> And borrowing dulls the edge of husbandry.
> This above all—to thine ownself be true;
> And it must follow as the night the day.
> Thou canst not then be false to any man."

Your intercourse with your professional brethren must be conducted upon the strictest principles of medical etiquette. Do you ask where you will find a complete code of Medical Ethics? I answer, in your own bosom, for there are none so ignorant as to be unacquainted with justice and injustice. There is but one code of ethics as there is but one geometry; a slight study of either compels us to draw the same conclusion. "That which you would that men should do unto you, do ye also unto them likewise," for that is all. Graduates! In behalf of the Dental Faculty of the University of the State of Iowa, I bid you an affectionate farewell.

